# Clinical, biochemical, and genetic spectrum of seven patients with *NFU1* deficiency

**DOI:** 10.3389/fgene.2015.00123

**Published:** 2015-04-13

**Authors:** Uwe Ahting, Johannes A. Mayr, Arnaud V. Vanlander, Steven A. Hardy, Saikat Santra, Christine Makowski, Charlotte L. Alston, Franz A. Zimmermann, Lucia Abela, Barbara Plecko, Marianne Rohrbach, Stephanie Spranger, Sara Seneca, Boris Rolinski, Angela Hagendorff, Maja Hempel, Wolfgang Sperl, Thomas Meitinger, Joél Smet, Robert W. Taylor, Rudy Van Coster, Peter Freisinger, Holger Prokisch, Tobias B. Haack

**Affiliations:** ^1^Institute of Human Genetics, Technische Universität MünchenMunich, Germany; ^2^Department of Pediatrics, Paracelsus Medical University of SalzburgSalzburg, Austria; ^3^Department of Pediatrics, Division of Pediatric Neurology and Metabolism, Ghent University HospitalGhent, Belgium; ^4^Wellcome Trust Centre for Mitochondrial Research, Institute of Neuroscience, Newcastle University Medical SchoolNewcastle upon Tyne, UK; ^5^Department of Clinical Inherited Metabolic Disorders, Birmingham Children’s HospitalBirmingham, UK; ^6^Department of Pediatrics, Technische Universität MünchenMunich, Germany; ^7^Division of Child Neurology, Children’s Research Center, Kinderspital ZürichZürich, Switzerland; ^8^Division of Metabolism, Children’s Research Center, Kinderspital ZürichZürich, Switzerland; ^9^Praxis für HumangenetikBremen, Germany; ^10^Research Group Reproduction and Genetics, Center for Medical Genetics, Vrije Universiteit Brussel, Universitair Ziekenhuis BrusselBrussels, Belgium; ^11^Elblab Zentrum für LaborMedizin, ElblandklinikenRiesa, Germany; ^12^Department of Pediatrics, Klinikum Bremen-MitteBremen, Germany; ^13^Institute of Human Genetics, University Medical Center Hamburg-EppendorfHamburg, Germany; ^14^Institute of Human Genetics, Helmholtz Zentrum MünchenNeuherberg, Germany; ^15^Department of Pediatrics, Klinikum ReutlingenReutlingen, Germany

**Keywords:** *NFU1*, iron–sulfur cluster, lipoic acid, mitochondrial respiratory chain, pulmonary hypertension

## Abstract

Disorders of the mitochondrial energy metabolism are clinically and genetically heterogeneous. An increasingly recognized subgroup is caused by defective mitochondrial iron–sulfur (Fe–S) cluster biosynthesis, with defects in 13 genes being linked to human disease to date. Mutations in three of them, *NFU1, BOLA3,* and *IBA57,* affect the assembly of mitochondrial [4Fe–4S] proteins leading to an impairment of diverse mitochondrial metabolic pathways and ATP production. Patients with defects in these three genes present with lactic acidosis, hyperglycinemia, and reduced activities of respiratory chain complexes I and II, the four lipoic acid-dependent 2-oxoacid dehydrogenases and the glycine cleavage system (GCS). To date, five different *NFU1* pathogenic variants have been reported in 15 patients from 12 families. We report on seven new patients from five families carrying compound heterozygous or homozygous pathogenic *NFU1* mutations identified by candidate gene screening and exome sequencing. Six out of eight different disease alleles were novel and functional studies were performed to support the pathogenicity of five of them. Characteristic clinical features included fatal infantile encephalopathy and pulmonary hypertension leading to death within the first 6 months of life in six out of seven patients. Laboratory investigations revealed combined defects of pyruvate dehydrogenase complex (five out of five) and respiratory chain complexes I and II+III (four out of five) in skeletal muscle and/or cultured skin fibroblasts as well as increased lactate (five out of six) and glycine concentration (seven out of seven). Our study contributes to a better definition of the phenotypic spectrum associated with *NFU1* mutations and to the diagnostic workup of future patients.

## Introduction

Combined defects of mitochondrial respiratory chain complexes are in the majority of cases associated with defects in the maintenance of the mitochondrial DNA, mitochondrial replication and translation, mitochondrial homeostasis and cofactor metabolism (Smits et al., 2010; Sperl et al., 2014). In case enzyme complexes beyond those in the oxidative phosphorylation (OXPHOS) system are impaired, it is unlikely that primary defects in mitochondrial DNA are responsible. Most likely, the enzyme impairment involves either synthesis or distribution defects of essential cofactors (Mayr et al., 2014; Desbats et al., 2015; Mayr, 2015). Iron–sulfur (Fe–S) clusters are one example of such a cofactor whose dysfunction results in multiple enzyme defects. A complex synthesis and distribution machinery is located within the mitochondria to sequester the various classes of Fe–S clusters to the enzymes which harbor them as essential cofactors (Lill et al., 2012). This machinery is called ISC (iron–sulfur cluster) assembly machinery. Two major types of ISC are assembled, the [2Fe–2S]- and [4Fe–4S]-clusters. The many steps involved in ISC assembly can be structured in three major steps: step 1 is the core ISC synthesis on a scaffold protein, step 2 is the release of the [2Fe–2S] clusters from the scaffold protein, and step 3 is the ISC targeting to the apoproteins with eventual transformation into [4Fe–4S] clusters. ISC are present in several essential protein complexes located in mitochondria. In the respiratory chain, complexes I, II, and III all contain subunits with ISC (Lill and Muhlenhoff, 2005). Furthermore, aconitase and lipoic acid synthetase (LIAS) contain ISC. LIAS is necessary for the synthesis of lipoic acid, a cofactor of the E2 subunit of the 2-oxoacid dehydrogenases such as pyruvate dehydrogenase (PDH) and the H-protein of the glycine cleavage system (GCSH; Mayr et al., 2014).

**FIGURE 1:**
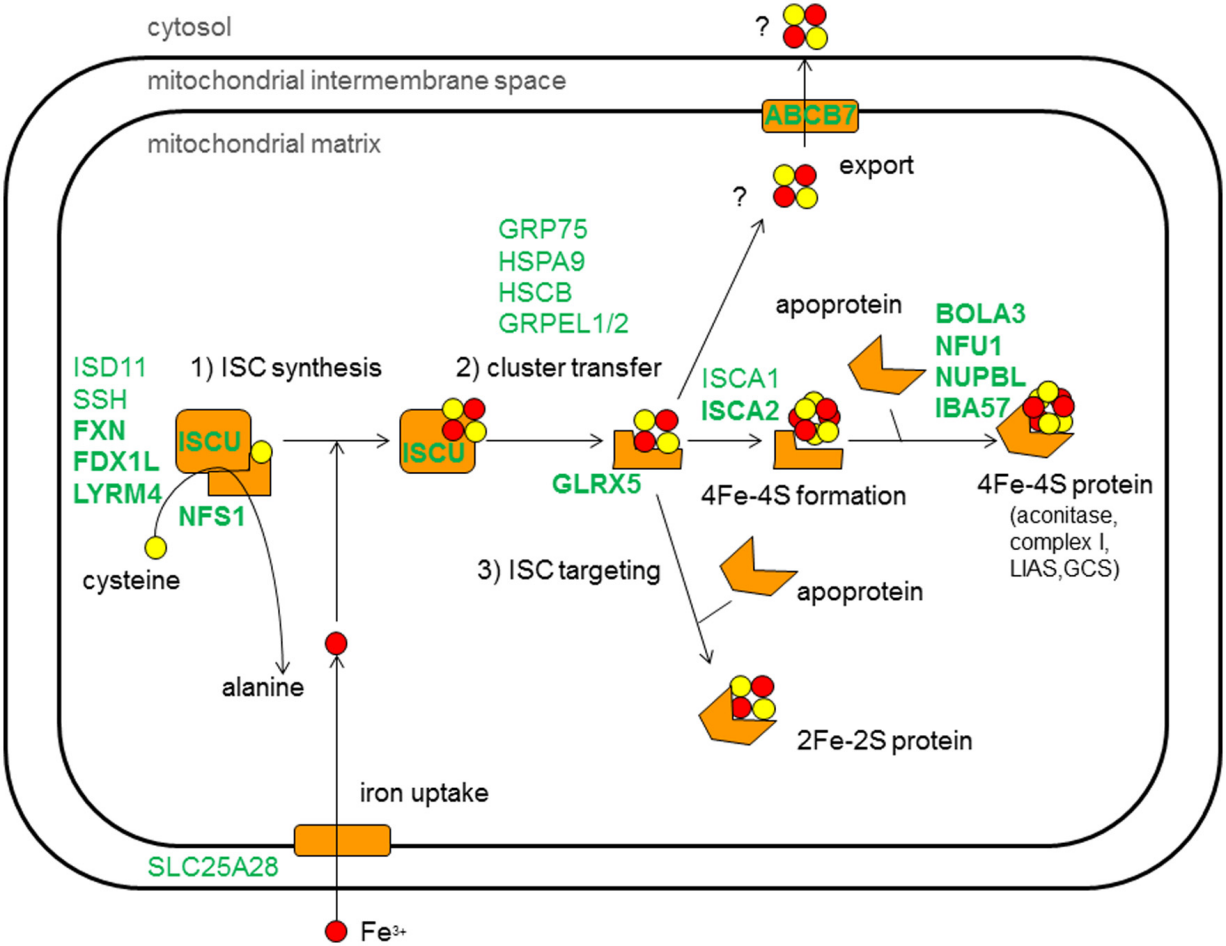
**Simplified schematic view of iron–sulfur cluster assembly machinery in mitochondria of human cells.** Known protein components are inticated in green, described according to its encoding gene. Disease-associated components are indicated in bold. Yellow spots represent sulfur molecules and red spots iron molecules. The molecules are assembled in [2Fe–2S]-cluster and [4Fe–4S]-clusters by the ISC machinery.

Experiments in yeast and human cell lines defined at least 17 components in the mitochondrial ISC assembly system (**Figure 1**). Associations with human disease have recently been reviewed by Stehling et al. (2014) and 13 of the ISC components have been linked to human disease presentations, including the core ISC assembly factors [FXN (Campuzano et al., 1996), ISCU (Mochel et al., 2008; Olsson et al., 2008), ISCA2 (Al-Hassnan et al., 2015), FDX1L (Spiegel et al., 2014), LYRM4 (Lim et al., 2013), and NFS1 (Farhan et al., 2014)] and factors involved in cluster transfer [GLRX5 (Camaschella et al., 2007; Baker et al., 2014)], the export of ISC [ABCB7 (Stehling and Lill, 2013)], maturation of mitochondrial complex I [NUBPL (IND1; Calvo et al., 2010)], mitochondrial iron import [SLC25A37 (MFRN1)], and three proteins necessary for targeting of the [4Fe–4S] clusters into apoproteins [IBA57 (Ajit Bolar et al., 2013), NFU1 (Cameron et al., 2011; Navarro-Sastre et al., 2011), and BOLA3 (Cameron et al., 2011; Haack et al., 2013)].

Genetic defects in *NFU1* (causing MMDS1, OMIM #605711), *BOLA3* (causing MMDS2, OMIM #614299), and *IBA57* (causing MMDS3, OMIM #615330), all factors acting in the final step of the assembly of [4Fe–4S] proteins, have been designated multiple mitochondrial dysfunction syndromes (MMDSs). Functional experiments led to the hypothesis that *NFU1* is involved in distribution of [4Fe–4S]-clusters to a subset of proteins including subunits of mitochondrial respiratory complexes I and II, and of LIAS (Navarro-Sastre et al., 2011). To our knowledge, at least 15 patients from 12 families carrying mutations in *NFU1* have been reported (Cameron et al., 2011; Navarro-Sastre et al., 2011; Invernizzi et al., 2014; Nizon et al., 2014). Key phenotypic features included failure to thrive, pulmonary hypertension, infantile encephalopathy, and neurological regression. Age of onset was 0–9 months and only two patients survived beyond the age of 15 months. A common founder mutation, c.622G>T, p.Gly208Cys, has been observed in ten Spanish and French patients and another four disease alleles have been reported in the remaining cases.

Here, we report the identification of clinically relevant compound heterozygous and homozygous DNA variants in *NFU1* in seven additional patients, and associated clinical and biochemical features.

## Patients, Materials, and Methods

### Case Reports

**FIGURE 2:**
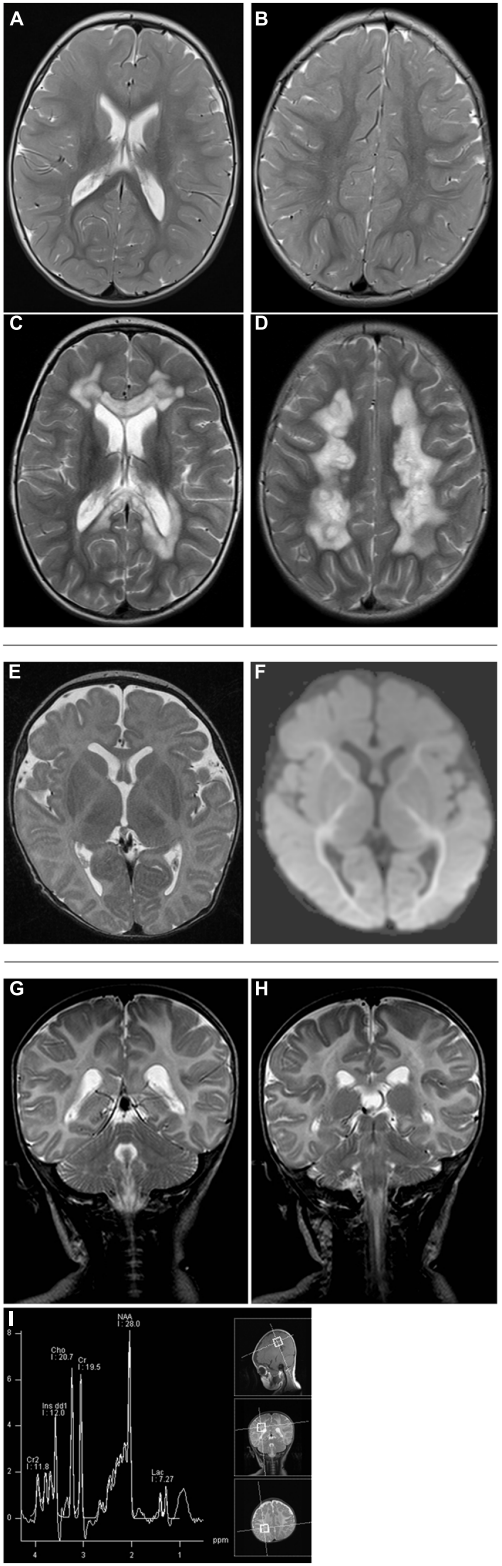
**Brain MRI studies in *NFU1*-mutant patients 2 [T2-weighted axial scans at age 1 2/12 years **(A,B)** and 2 2/12 years **(C,D)**], 3 [axial T2-weighted **(E)** and diffusion tensor image **(F)** at age 3 months], and 5 [T2-weighted coronar scans **(G,H)** and MR spectroscopy **(I)** at age 3 months].** A marked difference is seen in the extent and localization of the lesions ranging from progressive symmetric white matter lesions with necrotic regions in patient 2 **(C,D)** to reduced volume of supratentorial white matter **(E)** and diffusion restriction on DTI **(F)** and alterations in brainstem **(G)** and upper spinal cord **(H)**.

Written informed consent was obtained from all patients investigated or their guardians and the local ethics committees of the recruiting centers approved the study. Clinical and biochemical findings of *NFU1* mutation-positive patients are summarized in **Table 1** and abnormal MRI findings are shown in **Figure 2**.

**Table 1:** Genetic and phenotypic findings in *NFU1*-mutant individuals.

Patient ID	Sex	AO	AD	Identified variants		OXPHOS activities			Laboratory findings in plasma			Clinical features	
				Nucleotide	Amino acid	Enzyme	Muscle	Fibroblasts	Lactate (mmol/L)	Glycine (μmol/L)	MRI	Presenting	Additional
Patient 1	F	8 w	4 m	g.69400462C>A **g.69592691_** **69648327del**	p.Gly208Cys **?**	I II II+III IV PDHc	Normal 50% 43% Normal 69%	n.d. n.d. n.d. n.d. n.d.	25 (NR: 0.5-2.2)	Elevated	n.d.	Pulmonary arterial hypertension	Muscular hypotonia, apnea syndrome
Patient 2	M	23 w	30 m	c.565G>A **c.568G>A**	p.Gly189Arg **p.Gly190Arg**	I II+III IV PDHc	0% 25% 54% 20%	n.d. n.d. n.d. n.d.	2.4-4 (NR: 0.6-2.4)	670 (NR:120-386)	Leukoencephalopathy with marked periventricular involvement and partial necrosis	Pulmonary hypertension, muscular hypotonia	Swallowing difficulties, psychomotor regression
Patient 3	F	4 w	3 m	**c.544C>T** **?**	**p.Arg182Trp** **?**	I II II+III III IV PDHc	28% 0% n.d. Normal Normal n.d.	25% 30% 69% n.d. Normal 5%	11 (NR: 1-1.8)	636 (NR: 166-330)	Leukoencephalopathy affecting capsula interna, brainstem, hypomyelination, alterations in diffusion-weighted sequences	Apnea, bradycardia	Pulmonary arterial hypertension, muscular hypotonia, seizures
Patient 4^a^	F	9 w	3 m	**c.302+3A>G** **c.302+3A>G**	**p.Val56Glyfs^∗^9** **p.Val56Glyfs^∗^9**	I II+III IV PDHc	n.d. n.d. n.d. n.d.	Normal Normal Normal 35%	14 (NR 0.6-2.4)	576 (NR: 166-330)	n.d.	Failure to thrive, lactic acidosis	Acute dilated cardiomyopathy, pulmonary arterial hypertension
Patient 5^a^	M	11 w	3 m	**c.302+3A>G** **c.302+3A>G**	**p.Val56Glyfs^∗^9** **p.Val56Glyfs^∗^9**	I II III IV PDHc	n.d. n.d. n.d. n.d. n.d.	Normal Normal Normal Normal 30%	3.0 (NR 0.6-2.4)	1247 (NR: 166-330)	Leukoencephalopathy affecting upper cervical cord, dorsal medulla, inferior cerebellar peduncles, central hemispheric white matter and lack of normal myelination of the posterior limb of the internal capsule	Poor feeding, vomiting, failure to thrive	Developmental delay, muscular hypotonia and apnea, respiratory failure
Patient 6^a^	F	Birth	3 m	**c.302+3A>G** **c.302+3A>G**	**p.Val56Glyfs^∗^9** **p.Val56Glyfs^∗^9**	I-IV PDHc	n.d. n.d.	n.d. n.d.	n.d. (But moderately increased in urine organic acids)	1356 (NR: 166-330)	Alterations in diffusion-weighted sequences of internal and external capsules, brainstem, lentiform nuclei, and periventricular white matter	Poor feeding, vomiting, lactic acidosis, failure to thrive	Developmental delay, muscular hypotonia, apnea
Patient 7	M	10 w	5.5 m	**c.62G>C** c.622G>T	**p.Arg21Pro^b^** p.Gly208Cys	I II II+III III IV PDHc	Normal 15% 23% Normal 79% n.d.	n.d. 5% 10% Normal Normal n.d.	15 (NR: 1-1.78)	Elevated	n.d.	Failure to thrive, proximal tubulopathy, lactic acidosis	Muscular hypotonia, hypertrophic cardiomyopathy

M, male; F, female; m, months; w, weeks; OXPHOS, oxidative phosphorylation system; PDHc, pyruvate dehydrogenase complex; n.d., not determined; AO, age at onset; AD, age at death; NR, normal range; MRI, magnetic resonance imaging; ^a^these individuals are siblings. ^b^Sequence variant is predicted to interfere with splicing.

Reduced activity values of mitochondrial respiratory chain complexes I–IV (I–IV) are given normalized to citrate synthase (CS) activity in mUnit/mUnit CS in percent of the lower control range if not stated otherwise. Bold script indicates novel variants.

**Patient 1** (g.[69400462C>A];[69592691_69648327del], p.[Gl y208Cys];[?]), a girl, was the first child born to healthy non-consanguineous parents from Germany. After a normal pregnancy, she was born at 28 weeks of gestation with age-appropriate birth measurements (weight 1170 g, length 37 cm, head circumference 26.5 cm). She presented soon after birth with bradycardia and apnea requiring artificial ventilation. A persistent arterial duct was treated with Indomethacin. Her clinical condition thereafter stabilized although she continued to suffer from episodes of bradycardia and apnea. Echocardiogram performed at the age of 3 months revealed pulmonary arterial hypertension. At the age of 4 months she was re-admitted due to respiratory insufficiency. Serum lactate levels were persistently increased [up to 25 mmol/L; normal range (NR) 0.5–2.2 mmol/L] and her clinical condition worsened. Glycine was elevated in both urine (43734 μmol/L; NR <1240 μmol/L) and plasma. Biochemical analysis of skeletal muscle specimen showed decreased citrate synthase (CS)-adjusted activities of respiratory chain complexes II (0.09 mU/mU CS; NR 0.18–0.41 mU/mU CS), II+III (0.13 mU/mU CS; NR 0.30–0.67 mU/mU CS), and pyruvate dehydrogenase complex (PDHc; 0.018 mU/mU CS; NR 0.026–0.079 mU/mU CS). She died aged 4 months due to respiratory insufficiency and metabolic decompensation.

**Patient 2** (c.[565G>A];[568G>A], p.[Gly189Arg];[Gly190 Arg]), a boy, was the second child born to healthy non-consanguineous German parents. An older sister was similarly affected, a younger brother was healthy. After a normal pregnancy, he was born at 38 weeks of gestation with normal birth measurements (weight 3446 g, length 55 cm, head circumference 35 cm). Primary adaptation (Apgar 9/10/10) and the early development were normal. At the age of 23 weeks, muscular hypotonia and swallowing difficulties were noticed and subsequent diagnostic workup revealed pulmonary arterial hypertension. His further development was delayed and he developed spastic tetraparesis. At the age of 2 years he suffered from hematemesis and episodes of apnea. Ophthalmological examination revealed bilateral atrophy of the optic nerve. Laboratory investigations documented high normal lactate levels in serum (2.4 mmol/L; NR 0.6–2.4 mmol/L) and increased lactate concentration in capillary blood (4.0 mmol/L) and cerebrospinal fluid (CSF, 3.3 mmol/L; NR 1.2–2.1 mmol/L). Glycine levels were increased in both plasma (670 μmol/L; NR 120–386 μmol/L) and urine (6158 μmol/L; NR 110–645 μmol/L). Brain MRI performed at the age of 1 2/12 showed discrete signal T2-weighted hyperintense signal alterations in the peritrigonal region and in the right centrum semiovale (**Figures 2A,B**). Strikingly, brain MRI performed 1 year later at the age of 2 2/12 years showed marked symmetric T2-weighted hyperintensity in the white matter with partially necrotic regions (**Figures 2C,D**). The cerebellum, basal ganglia, and brainstem were unremarkable. Biochemical analysis in skeletal muscle showed decreased activities of all measured respiratory chain complexes (complex I: no detectable activity; NR 0.17–0.56 U/U CS; Complexes II+III: 0.02 U/U CS; NR 0.08–0.45 U/U CS; Complex IV: 0.6 mU/mU CS; NR 1.1–5.0 U/U CS) as well as PDHc (0.5 U^∗^100/U CS; NR 1.5–5.6 U^∗^100/U CS). His clinical condition declined with progressive neurological deterioration and he died aged 2 years and 5 months.

**Patient 3** (c.[544C>T];[?], p.[Arg182Trp];[?]), a girl, was the first child of healthy non-consanguineous parents from Serbia and Romania. After a normal pregnancy, she was born at 33 weeks of gestation with normal birth measurements (weight 2270 g, length 45 cm). Primary adaptation (Apgar 9/9/10) and early development were normal with visual contact and social smiling at the age of 8 weeks. From the age of 10 weeks failure to thrive, muscular hypotonia and episodes of intermittent bradycardia and apnea were noticed. A further diagnostic workup revealed pulmonary arterial hypertension as well as increased lactate concentration in plasma (up to 11 mmol/L, NR 1–1.8 mmol/L), urine (>10000 mmol/mol creatinine; NR 57–346 mmol/mol creatinine), and CSF (2.5 mmol/L; NR 1.1–1.7 mmol/L). In addition, glycine levels were markedly increased in plasma (636 μmol/L; NR 166–330 μmol/L) and CSF (39 μmol/L; NR 0.7–14 μmol/L). Brain MRI performed at the age of 3 months showed a leukodystrophy affecting the capsula interna and brainstem as well as diffuse hypomyelination and alterations of diffusion-weighted sequences (**Figures 2E,F**). Diffuse punctate bleeding was observed predominantly in the vermis. Biochemical analysis in a skeletal muscle specimen showed decreased CS-adjusted activities of respiratory chain complexes I (0.04 mU/mU CS; NR 0.14–0.28 mU/mU CS) and II (no detectable activity; NR 0.14–0.36 mU/mU CS). PDHc activity was not determined due to insufficient amount of biopsy material. Progressive neurological deterioration and respiratory insufficiency necessitated artificial ventilation and she died aged 3.5 months.

**Patients 4, 5, and 6** (c.[302+3A>G];[302+3A>G], p.[Val56 Glyfs^∗^9];[Val56Glyfs^∗^9])

**Patient 4** was the female child of first cousin consanguineous parents of Pakistani origin. She started showing failure to thrive at age of 9 weeks. She exhibited elevated lactate concentrations [highest 14 mmol/L in serum (NR 0.6–2.4 mmol/L), 7.3 mmol/L in CSF] and glycine was elevated in plasma (576 μmol/L; NR 166–330 μmol/L). Acute dilated cardiomyopathy was present which was progressive and had a fatal outcome at the age of 3 months due to severe pulmonary hypertension and right ventricular failure. Mildly increased intrafiber lipid was seen in post-mortem microscopic examinations of muscle and microvesicular steatosis was seen in liver. Liver transaminases were elevated (ALT 192 iU/L; NR 5–45, AST 358 iU/L; NR 0–80) but synthetic function was normal. Investigations in cultured fibroblasts demonstrated reduced PDH activity and abnormal long- and medium-chain fatty acid oxidation.

**Patient 5** was the male first cousin of patient four. He presented first with poor feeding, vomiting, mild lactate concentration elevation (up to 3.0 mmol/L in plasma and CSF), and failure to thrive. He also showed developmental delay, muscular hypotonia, and apnea. Glycine was elevated significantly in plasma (1247 μmol/L; NR 166–330 μmol/L). The brain MRI showed abnormal signal in the upper cervical cord, the dorsal medulla and inferior cerebellar peduncles as well as stippled signal change in the central hemispheric white matter and lack of normal myelination of the posterior limb of the internal capsule (**Figures 2G,H**). He died suddenly from an acute encephalopathy and respiratory failure at the age of 3 months. As seen in patient four, investigations in cultured fibroblasts demonstrated reduced PDH activity and abnormal long- and medium-chain fatty acid oxidation.

**Patient 6** was the later-born sibling of patient 5. She showed perinatal poor feeding, vomiting, lactic acidosis, and failure to thrive. In addition she showed developmental delay, muscular hypotonia, and apnea similar to her brother (patient 5). Invasive investigations were declined but glycine was elevated significantly [1356 μmol/L in plasma (NR 166–330 μmol/L), >3158 mmol/L in urine] and an early brain MRI showed increased signal on diffusion-weighted images in the posterior brainstem, internal and external capsules and lentiform nuclei. She had palliative care from birth and showed a rapid progression of disease, similar to her brother, dying aged 3 months.

**Patient 7** (c.[62G>C];[622G>T], p.[Arg21Pro];[Gly208 Cys]), a boy, was born to non-consanguineous parents from Belgium. Although presentation at birth was normal, at 10 weeks of age he presented with failure to thrive, lethargy, intermittent apnea, and stridor. He was hospitalized at the pediatric intensive care where initial evaluation revealed right ventricle heart decompensation, signs of tubulopathy, and hepatopathy with slight increase of liver enzymes. Biochemical workup showed elevated serum lactate (up to 15 mmol/L, NR 1–1.78 mmol/L), elevated glycine and lactate (4.8 mmol/L, NR 1.11–2.78 mmol/L) in CSF. Brain MRI performed shortly after hospitalization showed no abnormalities. Biochemical analysis of mitochondrial respiratory chain complexes normalized to CS activity (expressed as the logarithm of OXPHOS activity divided by the logarithm of CS activity. Deficient activities are considered when z-score: < -3.0) showed clear complex II deficiency in cultured skin fibroblast (z-score: -8.63), skeletal muscle homogenate (z-score -4.91), isolated mitochondria from skeletal muscle (z-score: -5.15), lymphocytes (z-score: -2.92), and liver tissue (z-score: -6.10). Additionally, activity of complex I was decreased in liver tissue (z-score: -3.58). PDHc activity has not been analyzed. Genetic workup for mtDNA alterations in skeletal muscle showed absence of MELAS (A3243G), MERRF (A8344G), NARP (T8993C/G) mutations. The patient’s condition deteriorated further and he died aged 4.5 months.

### Genetic Studies

#### Exome Sequencing

Exome sequencing and variant prioritization of patients 2 and 4 was essentially performed as described previously (Haack et al., 2014). A SureSelect Human All Exon 50 Mb V5 Kit (Agilent) was used for enrichment of coding DNA fragments. Sequencing was performed on a HiSeq2500 system (Illumina). BWA (version 0.5.87.5) was used for read alignment to the human reference assembly (hg19). Genetic variation was detected using SAMtools (v 0.1.18), PINDEL (v 0.2.4t), and ExomeDepth (v1.0.0). The average coverage was 128-fold and more than 97% of the target region was covered at least 20-fold allowing for high-confidence variant calls. Filtering of DNA variants was based on the assumption of a recessive mode of inheritance and focused on rare, non-synonymous and splice site variants affecting genes coding for mitochondrial proteins (Elstner et al., 2008).

#### Sanger Sequencing

Sanger sequencing was used to analyze the coding sequences and flanking intronic regions of *NFU1* in genomic DNA from patients 1, 3, and 7 and to confirm variants prioritized by exome sequencing (patients 2, 4, 5, and 6). Amplicons were stained with Serva DNA stain G (SERVA electrophoresis, Heidelberg) in 1% agarose gels, cycle-sequenced using BigDye chemistry 3.1, and run on an ABI 3130XL automatic sequencer (Applied Biosystems). Primer sequences and PCR conditions are available upon request. As reference sequence for *NFU1* cDNA we used NM_001002755.2 [the transcript containing a mitochondrial targeting sequence (predicted by http://ihg.gsf.de/ihg/mitoprot.html)] and for genomic DNA NC_000002.11.

### Biochemical Investigations

The enzymatic activities of respiratory chain complexes I–IV, PDHc, and CS were determined in muscle biopsy material from patients 1 and 3 as described previously (Strassburg et al., 2006; Feichtinger et al., 2010).

In patient 2 biochemical analyses were performed as described in the Supplemental Methods.

Biochemical investigations for patients 4, 5, and 7 were performed as described by Kirby et al. (2007) and Ajit Bolar et al. (2013).

### Quantification of Protein Levels

#### Immunofluorescence Studies

For double immunofluorescence staining the following antibodies were used: mouse monoclonal anti-complex II subunit SDHA (1:200; Abcam, Cambridge, UK), mouse monoclonal anti-VDAC1 (1:200; MitoSciences, Eugene, OR, USA), rabbit polyclonal anti-VDAC1 (1:200; Abcam, Cambridge, UK) and rabbit polyclonal anti-lipoic acid (1:200; Merck, Darmstadt, Germany). All primary antibodies were diluted in Dako antibody diluent with background reducing components (Dako, Glostrup, Denmark). The following secondary antibodies were used: anti-mouse Alexa Fluor 488 (1:500, Invitrogen, Eugene, OR, USA) and anti-rabbit Alexa Fluor 594 (1:1000, Invitrogen, Eugene, OR, USA). Secondary antibodies were diluted in PBS containing 0.5% Tween 20 (PBS-T, pH 7.4).

Cultured skin fibroblasts were grown on chamber slides overnight and washed in PBS twice the next day. Fixation was performed overnight at room temperature in 4% neutral buffered formaldehyde (VWR, Darmstadt, Germany) and washed in dH_2_O, followed by heat-induced epitope retrieval in EDTA-T buffer (1 mmol/L EDTA, pH 8.0, 0.05% Tween 20) for 40 min at 95°C. Sections were equilibrated with PBS-T before primary antibodies were applied for 1 h at room temperature. Afterward sections were washed three times in PBS-T and incubated for 1h with secondary antibodies. Specimens were again washed three times in PBS-T and incubated with 0.5 μg/ml DAPI (Sigma, St. Louis, MO, USA) for 10 min. Slides were washed twice in dH_2_O and mounted in Fluorescent Mounting Medium (Dako, Glostrup, Denmark).

#### Western Blot and BN-PAGE Analyses

Western blotting was performed as described by Ajit Bolar et al. (2013). Mitochondrial fractions were isolated from skeletal muscle and cultured skin fibroblasts. Proteins were solubilized and subsequently separated by tricine SDS PAGE. Western blotting was performed using a mixture of antibodies directed against one subunit in each of the five respiratory complexes: NDUFB8 for complex I, SDHB for complex II, core2 for complex III (UQCRC2), MT-CO2 for complex IV and subunit alpha for complex V (ATP5A1; MS601 Mito-Profile human total OXPHOS complexes detection kit, 1.5 mg/ml, MitoSciences, Eugene, OR, USA). Lipoic acid-containing proteins were detected by western blotting using an antibody against protein-bound LA (ab58724, Abcam, Cambridge, UK). Detection was achieved by using ECL Plus^TM^ enhanced chemiluminescence kit (GE Healthcare, Diegem, Belgium), as described previously (van der Westhuizen et al., 2010). A ChemiDoc charge-coupled device camera and Quantity One software was used for imaging (Bio-Rad, Nazareth, Belgium).

Blue native-PAGE was used to separate and visualize the respiratory chain complexes in mitochondria isolated from skeletal muscle. Solubilization of the complexes, BN-PAGE and staining of their in-gel catalytic activities were performed as reported (Van Coster et al., 2001). Patient and control samples were loaded in duplicate using equal amounts of mitochondrial proteins (∼50 μg).

## Results

### Genetic and Biochemical Results

**FIGURE 3:**
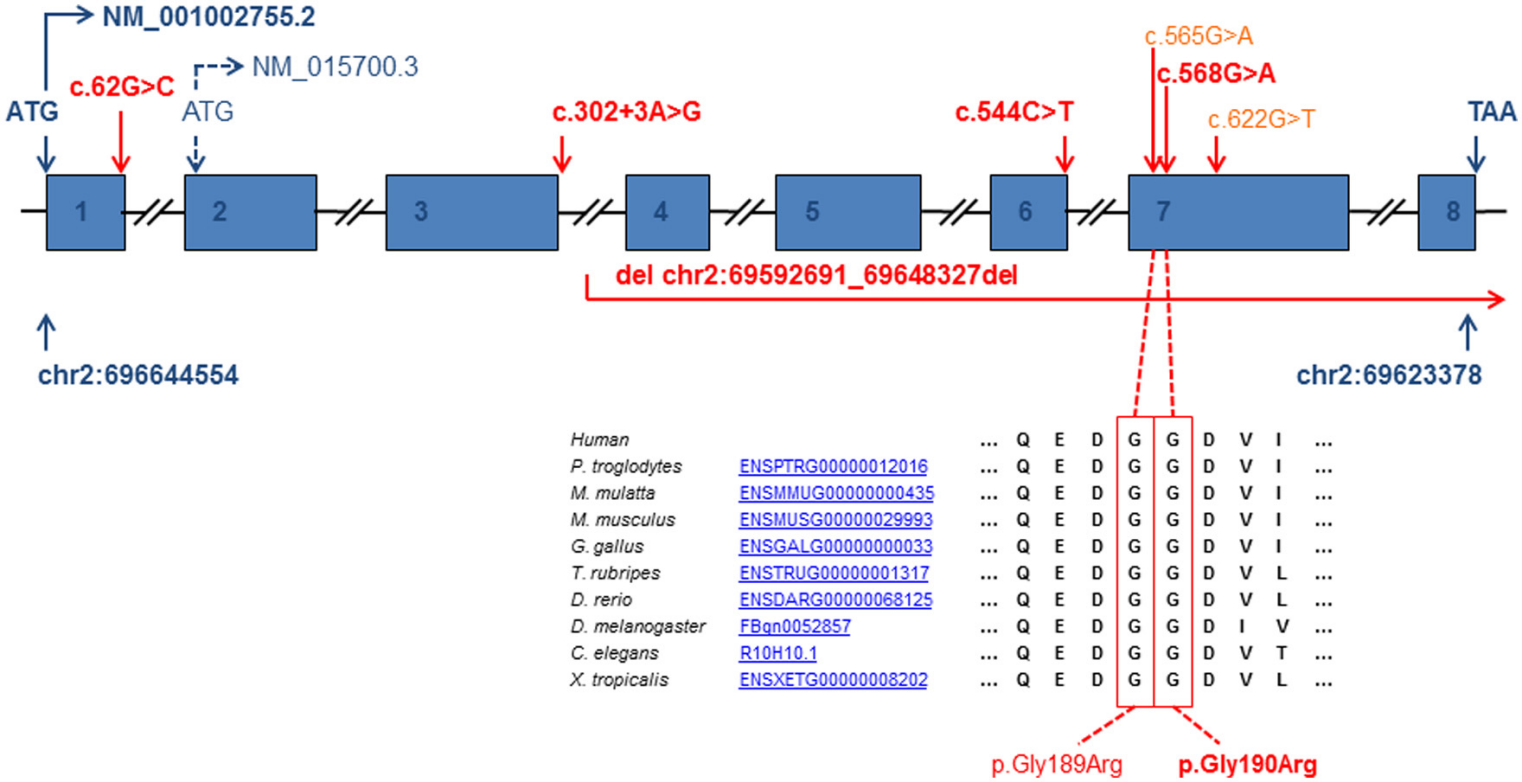
**Gene structure of *NFU1* and position of the identified pathogenic variants (reference cDNA sequence NM_001002755.2 and genomic DNA of chromosome 2 NC_000002.11).** Bold script indicates newly identified pathogenic variants. Introns are not drawn to scale.

Exome sequencing and candidate gene sequencing identified a total of seven different disease alleles (**Figure 3**) in five families and one yet to be fully defined change leading to a lack of mature mRNA expression. The observed mutations include two previously described missense mutations (c.565G>A, p.Gly189Arg and c.622G>T, p.Gly208Cys), three novel missense mutations (c.62G>C, p.Arg21Pro; c.544C>T, p.Arg182Trp and c.568G>A, p.Gly190Arg), one splice site mutation predicting a truncated protein (c.302+3A>G, p.Val56Glyfs^∗^9), and one contiguous gene deletion including coding exons 4–8 of NFU1. Carrier testing of available parental samples confirmed a biallelic localization of the identified NFU1 variants.

**FIGURE 4:**
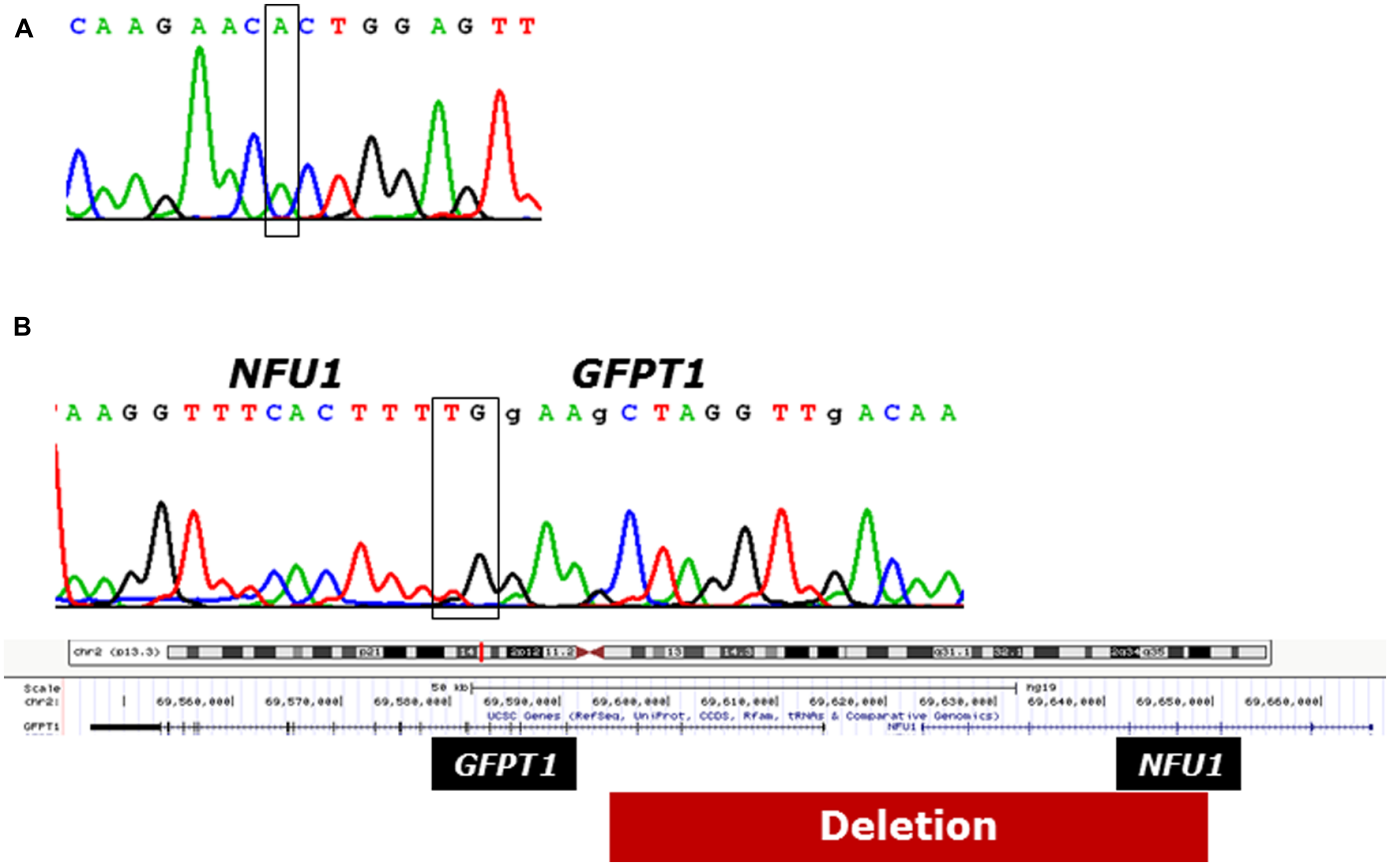
**Genetic analysis of patient 1. (A)** Electropherogram of the strand of cDNA of *NFU1*. The box indicates the position of pathogenic variant c.622G>T. **(B)** Break point analysis of the deletion on the non-expressed allele of *NFU1*.The box above the electropherogram indicates the breakpoint; below illustrates the position of the deletion in the chromosome.

In patient 1 the previously reported founder mutation c.622G>T, p.Gly208Cys in exon 7 was identified in apparently homozygous state. However, further experiments showed that the other allele carried a 55.6 kb deletion (hg 19, chromosome 2:69592691 – 69648327) affecting exons 4–8 of NFU1 and exons 1–3 of neighboring GFPT1 (**Figure 4**). Mutations in GFTP1, coding for glutamine:fructose-6-phosphate amidotransferase, have been associated with an autosomal-recessive congenital myasthenic syndrome with tubular aggregates, 1 (CMSTA1; OMIM #610542). We found no evidence of another potentially pathogenic GFPT1 allele for patient 1, suggesting that the identified biallelic NFU1 mutations are likely to underlie his clinical presentation.

Patient 2 carried a previously reported missense variant (c.565G>A, p.Gly189Arg) compound heterozygous with a novel missense variant affecting the neighboring amino acid c.568G>A, p.Gly190Arg. Both amino acids are conserved in several species (**Figure 3**) and are predicted to have a pathogenic impact on protein function by several programs (PolyPhen2, SIFT, MutationTaster).

**FIGURE 5:**
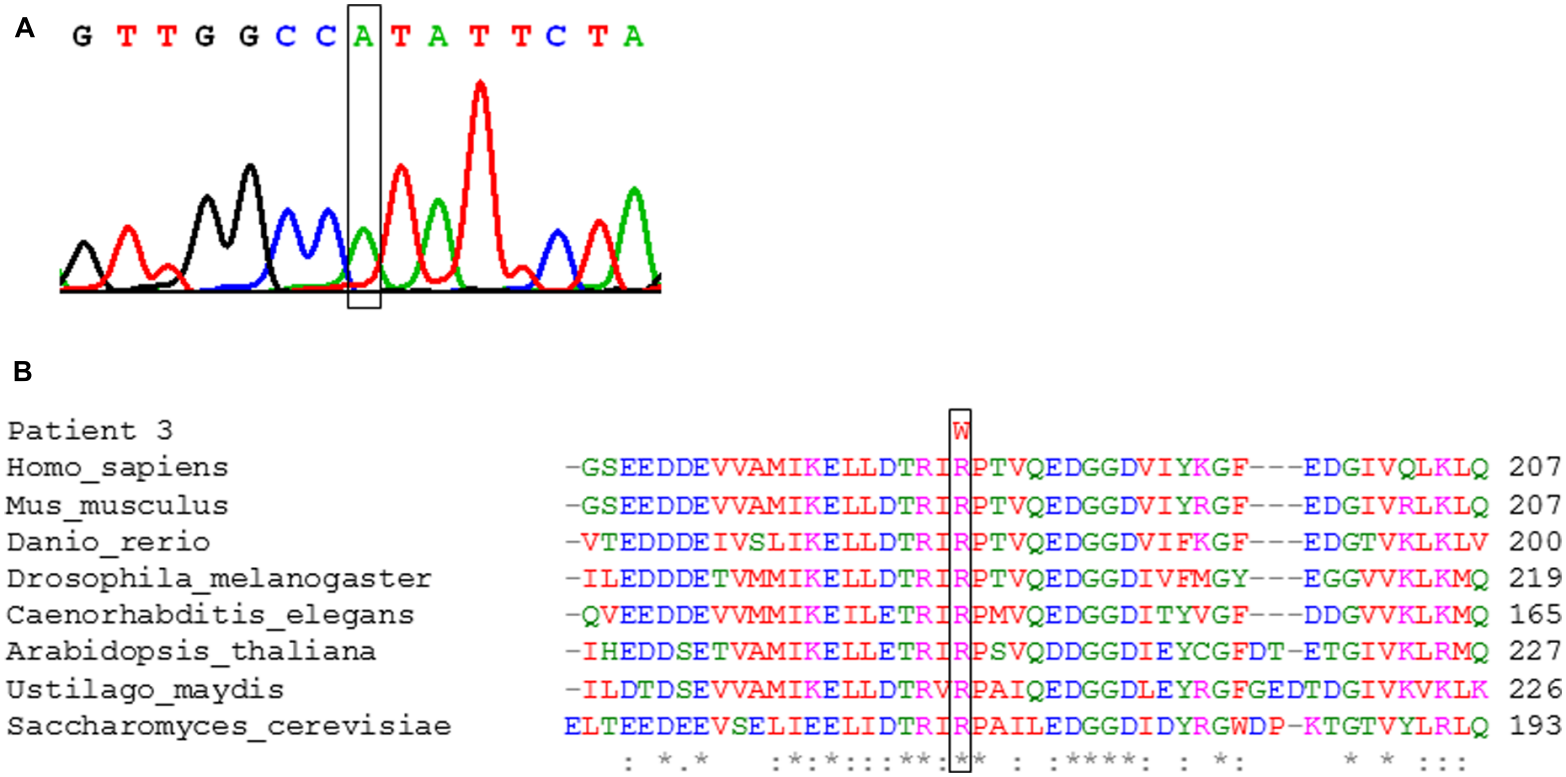
**Genetic analysis of patient 3. (A)** Electropherogram of the strand of cDNA of *NFU1*. The box indicates the position of the pathogenic c.544C>T variant. **(B)** Conservation of the amino acid residue at position 182 affected by the pathogenic c.544C>T, p.Arg182Trp variant in the indicated species. The box marks the changed position.

In patient 3, a single novel missense mutation (c.544C>T, p.Arg182Trp) was identified in the heterozygous state leading to a change affecting a highly conserved residue in the polypeptide chain (**Figure 5B**). The same amino acid is affected by the reported variant c.545G>A, p.Arg182Gln and has been shown to affect mRNA splicing (Cameron et al., 2011). Further investigations showed that in the processed mRNA only the c.544C>T was detectable in cultured skin fibroblasts from the patient (**Figure 5A**). These findings suggest a yet to be defined loss-of-function mutation on the second allele.

Patients 4–6 from consanguineous family 4 carried the same homozygous mutation c.[302+3A>G];[302+3A>G], p.[Val56Glyfs^∗^9];[Val56Glyfs^∗^9]. *In silico* splice-site prediction tools predicted that the c.302+3A>G substitution is likely to significantly reduce the efficiency of the consensus donor splice site of intron 3, with the most likely effect being skipping of exon 3 during splicing. Total RNA was isolated from patient fibroblasts and RT-PCR performed using primers designed to flank exon 3. We observed a significant increase in the proportion of *NFU1* transcripts (both cytosolic- and mitochondrial-specific isoforms) lacking exon 3 as compared to tissue-matched normal controls, confirming that the c.302+3A>G substitution leads to aberrant splicing of *NFU1* transrcipts (data not shown). The predicted effect of exon 3 skipping at the protein level is an out-of-frame deletion leading to the introduction of a premature termination codon 8 positions downstream, p.Val56Glyfs^∗^9.

Patient 7 carried a previously described missense (c.622G>T, p.Gly208Cys) mutation compound heterozygous with a novel mutation c.62G>C. This mutation predicts an amino acid change p.Arg21Pro in the translated protein. However, as it affects the last base pair of exon 1 and is predicted to significantly reduce the efficiency of the consensus donor splice site of exon 1, it may also cause a splice defect. This variant underlines the functional relevance of the long isoform (NM_001002756.2) containing a predicted mitochondrial targeting sequence over the shorter isoform (NM_015700.3) resulting from an alternative start codon located in exon 2. All *NFU1* variants are very rare and absent from the ExAC browser with exception of the c.622G>T, p.Gly208Cys change which has been detected 16 times in the heterozygous state in 122,946 control alleles (MAF 0.013%; Exome Aggregation Consortium (ExAC), Cambridge, MA, USA (URL: http://exac.broadinstitute.org) [11/2014]).

**FIGURE 6:**
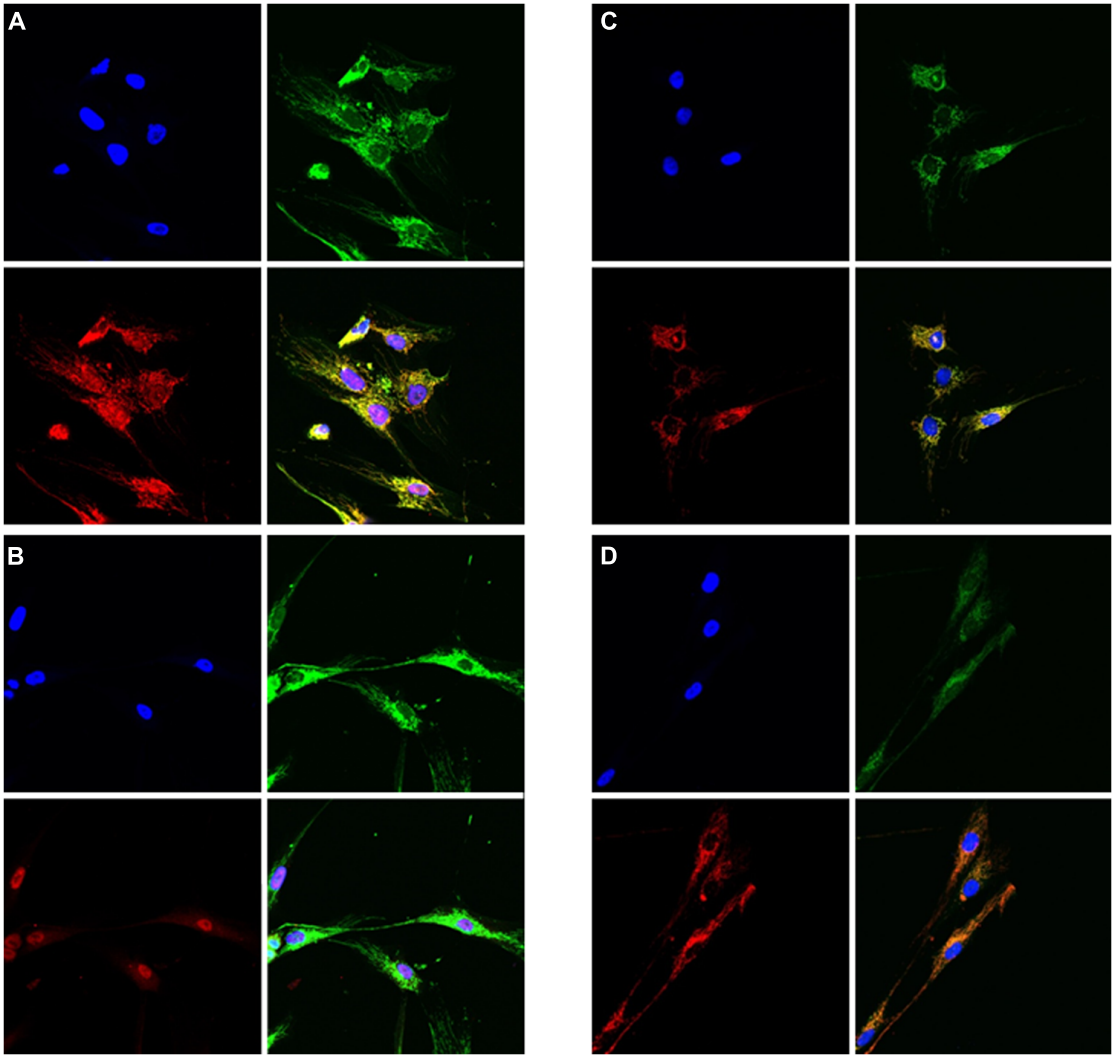
**Immunofluorescence studies of cultured skin fibroblast cells from patient 3 and a control showing reduced lipoic acid content and reduced amount of SDHA in the patient cells as compared to controls. (A,B)** Staining on lipoic acid; A = control cells, B = patient cells. Blue = DAPI-stain (nuclei), green = porin, red = lipoic acid. **(C,D)** Staining on SDHA; C = control cells, D = patient cells. Blue = DAPI-stain (nuclei), red = porin, green = SDHA. In all **(A–D)** the lower-right picture shows the merge of the other three pictures.

In patients 3 and 7 functional studies were performed to investigate the cellular consequences of NFU1 deficiency. Defective [4Fe–4S] incorporation is expected to impair proper assembly of respiratory chain complexes and LIAS. In fibroblast cell lines from patient 3 (c.[544C>T];[?], p.[Arg182Trp];[?]) we therefore performed immunohistochemical staining for a subunit of complex II and lipoic acid. Levels of both were found to be severely decreased in NFU1-mutant cell lines as compared to controls (**Figure 6**).

**FIGURE 7:**
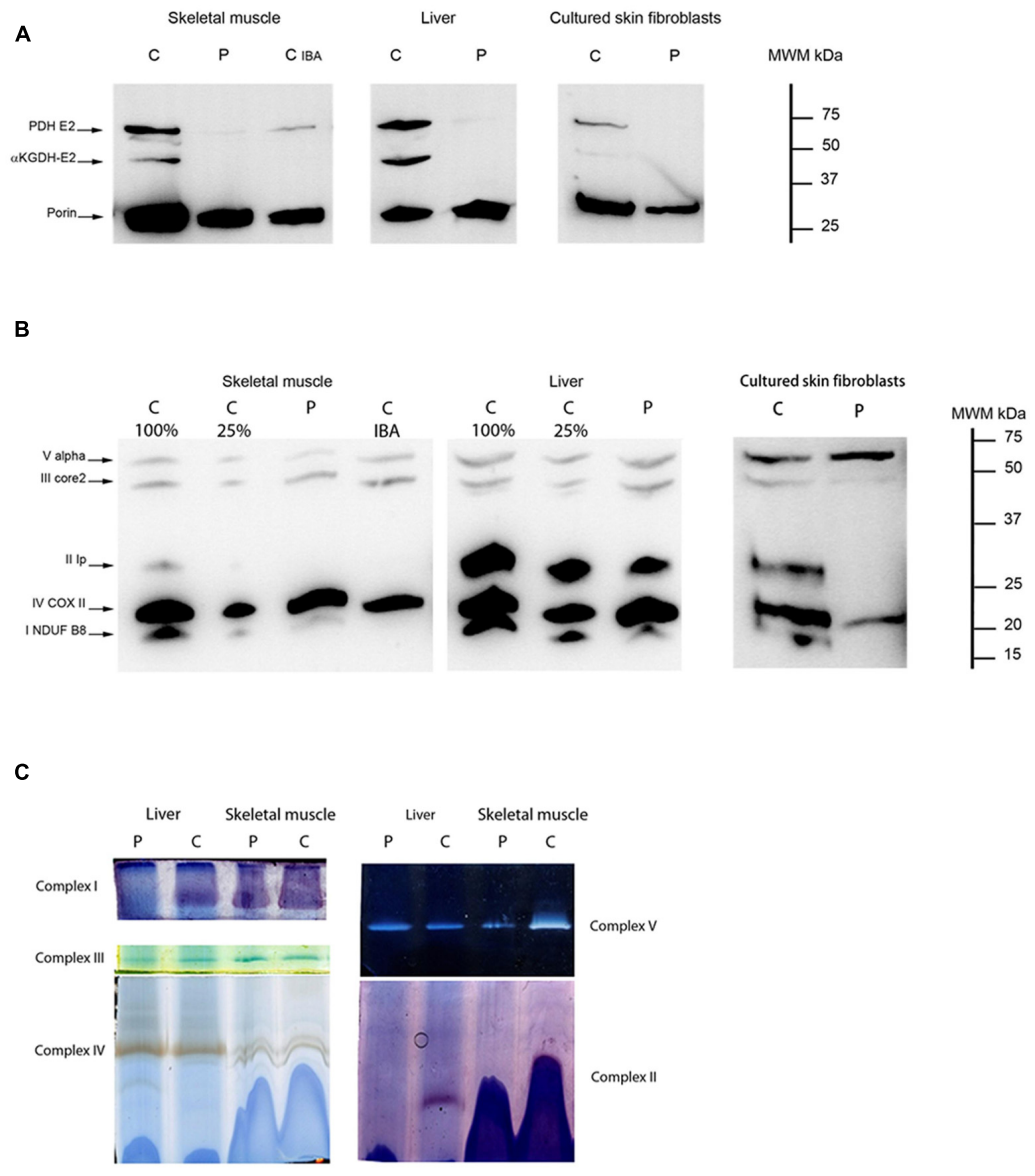
**Lipoic acid loading and OXPHOS complex expression in patient 7. (A)** Western blot showing expression levels of lipoic acid residues of subunit E2 of PDH (PDH E2) and subunit E2 of αKGDH (αKGDH-E2) in skeletal muscle, liver, and cultured skin fibroblast for patient 7 (p) compared to a control sample (c). The mitochondrial protein VDAC1 (porin) was used as loading control. Together with both skeletal muscle samples of control and patient, a patient with a pathogenic mutation in *IBA57* (c_IBA_) was loaded in parallel to illustrate the similarity of defective lipoylation. **(B)** Western blot showing expression levels of different subunits of all five OXPHOS complexes (NDUFB8 for complex I, Ip for complex II, core2 for complex III, COXII for complex IV, and Valpha for complex V) in skeletal muscle, liver, and cultured skin fibroblasts in patient 7 (p) and a control sample (c). There is absence of CRM-signal for complex II subunit in skeletal muscle and cultured skin fibroblasts. In liver the CRM-signal for complex II is almost equal to the control loaded at 25% (c25%). CRM-signal for complex I is undetectable (skin fibroblasts) or lower than c25% (skeletal muscle and liver). For skeletal muscle a patient with a pathogenic mutation in *IBA57* (c_IBA_) was loaded in parallel to illustrate the similarity of lowered OXPHOS subunit expression. **(C)** Blue native-PAGE with in-gel activity staining is shown for liver (left side) and skeletal muscle (right side) in patient 7 (p) and a control sample (c) illustrating severely decreased complex I in liver and complex II activity in both tissues. Complex I activity in skeletal muscle is partially decreased. Samples are loaded in duplo and one gel is used for in-gel activity staining of complex I, III and IV (left panel) while the other is used for complexes V and II (right panel).

In patient 7, we investigated the amount and function of lipoic acid-containing enzyme complexes in skeletal muscle, cultured skin fibroblasts and liver. The E2 subunit of PDHc and E2 subunit of α-ketoglutarate dehydrogenase were virtually absent in all patient tissues analyzed (**Figure 7A**). BN-PAGE separation of OXPHOS complexes followed by in-gel activity staining in skeletal muscle and liver tissue revealed a clear complex II deficiency (**Figure 7B**). Evaluation of protein amount in skeletal muscle and liver tissue were concordant with BN-PAGE findings, with absence of cross-reacting material for Fe–S and Fp subunit of complex II (**Figure 7C**) as well as lowered abundance of complex I. These findings could also be demonstrated in cultured patient skin fibroblasts. Together, these findings are consistent with a pathogenic role of NFU1 mutations identified in patients 3 and 7.

## Discussion

The phenotypic spectrum associated with *NFU1* mutations has been recently reviewed by Invernizzi et al. (2014). Key phenotypic features observed in the 15 reviewed patients included failure to thrive, pulmonary hypertension, infantile encephalopathy, and neurological regression. Age of onset was 0–9 months and only two patients survived beyond the age of 15 months. Notably, these two patients reported by Invernizzi et al. (2014) and Nizon et al. (2014) carried the c.565G>A, p.Gly189Arg missense mutation on one allele. The same mutation has been also identified in patient 2 who survived until the age of 30 months while all other six patients in our cohort died within the first half year of life. It can be speculated that, besides being the second most common *NFU1* mutation, the c.565G>A, p.Gly189Arg change might be associated with a slightly milder course of the disease.

By far the most common mutation in *NFU1* is a c.622G>T, p.Gly208Cys founder mutation observed in ten Spanish and French patients and in compound heterozygosity with other mutations in patients 1 and 7 in this present work. It has a MAF of 0.013% in the ExAC Browser [Cambridge, MA, USA (URL: http://exac.broadinstitute.org) [11/2014]) and is therefore likely to be amongst the more common causes of mitochondrial genetic disease at least in certain populations.

The c.62G>C, p.Arg21Pro mutation found in patient 7 provides an evidence that the mitochondrial form of *NFU1*, represented by the transcript NM_001002755.2, is the relevant isoform for *NFU1* deficiency, which is biochemically and functionally clearly a mitochondriopathy.

All seven patients presented with infantile encephalopathy characterized by muscular hypotonia, psychomotor developmental delay, and neurological regression. Additional clinical features included episodes of apnea and bradycardia. Pulmonary hypertension has been documented in four out of seven patients and two patients died from acute cardiac decompensation. Additional findings included poor feeding, vomiting and signs of renal and hepatic involvement.

In 5 out of 15 published cases neuroimaging data were available and it has been suggested that signs of leukoencephalopathy in the periventricular white matter and corpus callosum with partial cystic degeneration and cavitations, described in more detail in two patients, could be a specific MRI pattern related to MMDS1 (Invernizzi et al., 2014; Nizon et al., 2014). In the latter two patients, the basal ganglia, cerebellum, and brain stem were normal.

Brain MRI data were available from five patients in this cohort. Four of them had a leukodystrophy in the course of the disease while in patient 7 no abnormalities had been observed at the age of 10 weeks. The extent and localization of the observed lesions was variable. In patient 2, the first MRI performed at the age of 1 2/12 years showed only mild alterations while a second MRI performed 1 year later showed severe leukoencephalopathy with involvement of the periventricular white matter and corpus callosum with partial necrotic changes. Together with the normal appearance of other structures including brainstem, cerebellum and basal ganglia, this neuroimaging pattern is in line with the findings of the two patients reported by Invernizzi et al. (2014) and Nizon et al. (2014). However, the documented progress of the brain lesions observed within 1 year in patient 2 also demonstrates that even in clinically severely affected individuals MRI lesions can be mild at an early stage of the disease and that cystic degeneration might only evolve at a later stage in longer-surviving patients. Furthermore, our findings in the three other patients indicate marked differences in extent and localization of the lesions. Besides central hemispheric white matter, affected structures included capsula interna and externa, lentiform nuclei, brainstem, upper cervical cord, and inferior cerebellar peduncles.

The signature of abnormal metabolites in published cases included increased levels of lactate and glycine in plasma, urine, and CSF as well as increased urinary excretion of TCA cycle intermediates. Variable lactic academia was observed in all seven patients of our cohort as well as elevation of glycine in plasma (7/7), CSF (2/2), and urine (3/3).

Pyruvate dehydrogenase complex activity so far had been assessed in frozen muscle of two patients (Invernizzi et al., 2014; Nizon et al., 2014) and showed clear reduction to <50% of the lower control value. This corresponds to the measurements of PDHc activity in skeletal muscle of patients 1 and 2 showing a reduction to 30 and 69% of the lowest control value, respectively. Activities of mitochondrial respiratory chain complexes have so far been investigated in four published cases. Only one out of three patients investigated by Navarro-Sastre et al. (2011) had an abnormal finding with activities for complex II+III just below the control range and in one patient reported by Invernizzi et al. (2014), complex II was decreased to 67% of the lowest control value and complex I was just below normal. Skeletal muscle specimen analyzed in four patients in the present cohort demonstrated a consistent decrease of complex II and complexes II+III activity to 0–60% of the lowest control value. Complex I activity was decreased in skeletal muscle of patients 2 and 3 to 0–58% of controls and in a liver biopsy of patient 7. Furthermore, complex IV activity was found below the normal range in two out of four individuals. Investigation in fibroblasts performed in patients 3 and 7 showed the similar patterns as in skeletal muscle for complex I (patient 3) and complex II (patients 3 and 7) while the analysis in fibroblast of patient 5 indicated normal activities. Taken together, in *NFU1* patients analyses of mitochondrial respiratory chain complexes I–IV showed variable findings even for the same tissues being analyzed. However, investigation of PDHc activity showed a clear decrease in both *NFU1-*mutant fibroblasts (13 out of 13) and skeletal muscle (four out of four) and thus seems to be a consistent marker in *NFU1* deficiency.

In summary, we report the clinical, biochemical and genetic data on seven new patients with MMDS1 caused by *NFU1* mutations, six of which were novel genetic variants. The fact that one of them was a deletion of several exons underlines the necessity for deletion screening in molecular diagnostics of suspected *NFU1* deficiency. The observed phenotypic hallmarks of increased glycine concentrations in plasma and PDHc deficiency confirm findings of previous studies and may point toward a targeted analysis of potential causes of Fe–S clusters/lipoic acid biosynthesis defects. Although not necessarily persistent and only observed in 50% of cases, pulmonary hypertension in infancy might be an additional clinical feature to prioritize testing of *NFU1* amongst several other candidate genes.

## Conflict of Interest Statement

The authors declare that the research was conducted in the absence of any commercial or financial relationships that could be construed as a potential conflict of interest.
