# Supplementary material for: Clinical, biochemical, and genetic spectrum of seven patients with NFU1 deficiency
Source: Front Genet. 2015 Apr 13;6:123. doi: 10.3389/fgene.2015.00123 (PMC4394698; doi:10.3389/fgene.2015.00123)
Supplement: Supplementary file 1 [file Data_Sheet_1.DOCX]

***Supplementary Material***

**Uwe Ahting^1^, Johannes A. Mayr^2^, Arnaud V. Vanlander^3^, Steven A. Hardy^4^, Saikat Santra^5^, Christine Makowski^6^, Charlotte L. Alston^4^, Franz A. Zimmermann^2^, Lucia Abela^7^, Barbara Plecko^7^, Marianne Rohrbach^8^, Stephanie Spranger^9^, Sara Seneca^10^, Boris Rolinski^11^, Angela Hagendorff^12^, Maja Hempel^13^, Wolfgang Sperl^2^, Thomas Meitinger^1,14^, Joél Smet^3^, Robert W. Taylor^4^, Rudy Van Coster^3^, Peter Freisinger^15^, Holger Prokisch^1,14^, Tobias B. Haack^1,14,*^**

^1^Institute of Human Genetics, Technische Universität München, Munich, Germany

^2^Department of Pediatrics, Paracelsus Medical University Salzburg, Salzburg, Austria

^3^Department of Pediatrics, Division of Pediatric Neurology and Metabolism, University Hospital Ghent, Ghent, Belgium

^4^Wellcome Trust Centre for Mitochondrial Research, Institute of Neuroscience, The Medical School, Newcastle University, Newcastle upon Tyne, United Kingdom

^5^Department of Clinical Inherited Metabolic Disorders, Birmingham Children's Hospital, Birmingham, United Kingdom^6^Department of Pediatrics, Technische Universität München, Munich, Germany

^7^Devision of Child Neurology, Kinderspital Zürich, Zürich, Switzerland

^8^Division of Metabolism, Children’s Research Center, Kinderspital Zürich, Zürich, Switzerland

^9^Praxis für Humangenetik, Bremen, Germany

^10^Center for Medical Genetics, UZ Brussel, Vrije Universiteit Brussel (VUB); Research Group Reproduction and Genetics, Vrije Universiteit Brussel (VUB), Brussels, Belgium

^11^Elblab Zentrum für LaborMedizin, Elblandkliniken, Riesa, Germany

^12^Department of Pediatrics, Klinikum Bremen Mitte, Bremen, Germany

^13^Institute of Human Genetics, University Medical Center Hamburg-Eppendorf, Hamburg, Germany

^14^Institute of Human Genetics, Helmholtz Zentrum München, Munich, Germany

^15^Department of Pediatrics, Klinikum Reutlingen, Reutlingen, Germany

***Correspondence:** Tobias Haack, Institute of Human Genetics, Technische Universität and Helmholtz Zentrum München, Trogerstr. 32, 81675 Munich, Germany

tobias.haack@helmholtz-muenchen.de

1. **Supplementary Methods**

Biochemical analyses performed in patient 2 were performed as follows:

Approximately 30 mg of frozen muscle was cut in small pieces and mixed with 600 µl of SETH buffer (250 mmol/L saccharose, 2 mmol/L EDTA, 50 U/l heparin, 10 mmol/L Tris/HCl, pH 7.4). Homogenization was done in an ice bath with a 2 ml Teflon potter (20 strokes, rotation 1600 U/min). The homogenate was centrifuged for 15 min at 600g at 4°C. This 600 g supernatant was stored on ice until analysis. Non collagen protein in the homogenate was determined according to Lowry (Lowry, Rosebrough et al. 1951). Complex I (NADH coenzyme Q oxidoreductase) activity measurement: 480 µl reaction mix (30 mmol/L potassium phosphate, pH 7.4, 30 mg/ml BSA, 5 mmol/L MgCl_2_, 200 µM NADH, 8 µg/ml antimycin A) was supplemented by 15 µl decylubiquinon (1 mg/100 µl ethanol + 900 µl H_2_O) and pre-incubated 6 min at 30°C. The reaction was started by addition of 20 µl 600 g supernatant. The absorption was measured for 2 minutes at 334 nm. After addition of 2 µl rotenone (2 mg/ml ethanol) absorption was further monitored for 2 minutes to measure the complex I-independent NADH dehydrogenase activity. Complex II+III (succinate cytochrome c oxidoreductase) activity measurement: To start the reaction 20 µl 600 g supernatant was added to 480 µl reaction mix (16 mmol/L potassium phosphate, pH 7.5, 1,6 mmol/L EDTA, 1,6 mmol/L NaN_3_, 2 mg/ml oxidized cytochrome c, 2.4 mmol/L succinate, 4 µM rotenone) at 30°C. Absorption was measured for 4 minutes at 550 nm. Complex IV (cytochrome c oxidase) activity measurement: To start the reaction 10 µl 600 g supernatant was added to 480 µl reaction mix (10 mmol/L potassium phosphate, pH 7.0, 80 µM reduced cytochrome c) at 30°C. Absorption was measured for 2 minutes at 550 nm. For complete oxidation of cytochrome c sodium hexacyanoferrate(III) was added. Citric acid synthase activity measurement: 10 µl 600 g supernatant was incubated for 2 min with 500 µl reaction mix (90 mmol/L TRAM, pH 8.5, 3.3 mmol/L malate, 240 µM 3-acetylpyridine adenine dinucleotide, 30 µg/ml malate dehydrogenase) at 37°C. The reaction was started by addition of 10 µl acetyl CoA (10 mg/ml) and absorption was measured for 2 min at 366 nm. Pyruvate dehydrogenase (PDH) activity measurement: 40 µl of muscle homogenate was added to 360 µl reaction mix (5 mmol/L MgSO_4_, 2.5 mmol/L CaCl_2_, 625 µM NADH, 0.625 mmol/L EDTA, 1.25 mmol/L thiamine pyrophosphate, 250 µM coenzyme A, 6.25 mmol/L carnitine hydrochloride, 125 mmol/L Tris/HCl, pH 7.4) and incubated for 5 minutes at 37°C. The reaction was started by addition of 100 µl pyruvate (0.25 mol/L sodium pyruvate + 12.5 nCi [1-^14^C]-pyruvate in 2.5 mmol/L acetic acid) in airtight tubes and incubated for 20 minutes. The reaction was stopped by addition of 200 µl of 3 M HClO_4_. Released ^14^CO_2_ was trapped in 200 µl hyamine present in a separate tube placed in each of the airtight tubes before the reaction was started. Disintegrations per minute of ^14^C were determined in a ß-counter (Wallac). Blank value was subtracted from the samples value. Control range was established by measurement of skeletal muscle probes of a cohort of healthy adult individuals. The range was not validated for patients from birth up to an age of 3 months because of a lack of samples of healthy individuals of this age.

1. **Supplementary References**
2. Lowry, O. H., N. J. Rosebrough, A. L. Farr and R. J. Randall (1951). "Protein measurement with the Folin phenol reagent." J Biol Chem **193**(1): 265-275.
